# Equitable access to integrated primary mental healthcare for people with severe mental disorders in Ethiopia: a formative study

**DOI:** 10.1186/s12939-016-0410-0

**Published:** 2016-07-26

**Authors:** Maji Hailemariam, Abebaw Fekadu, Medhin Selamu, Girmay Medhin, Martin Prince, Charlotte Hanlon

**Affiliations:** 1 College of Health Sciences, School of Medicine, Department of Psychiatry, Addis Ababa University, Addis Ababa, Ethiopia; 2 Psychology and Neuroscience, Department of Psychological Medicine, Centre for Affective Disorders, King’s College London, Institute of Psychiatry, London, UK; 3 Aklilu Lemma Institute of Pathobiology, Addis Ababa University, Addis Ababa, Ethiopia; 4 Population Research Department, Centre for Global Mental Health, King’s College London, Institute of Psychiatry, Psychology and Neuroscience, Health Services, London, UK

**Keywords:** Ethiopia, Access to care, Barriers to access, Mental health care, Primary care, Psychosis

## Abstract

**Background:**

The provision of mental healthcare through integration into primary care is expected to improve access to care, but not all population groups may benefit equally. The aim of this study was to inform delivery of a new primary care-based mental health service in rural Ethiopia by identifying potential barriers to equitable access to mental healthcare and strategies to overcome them.

**Methods:**

A qualitative study was conducted as formative work for the PRogramme for Improving Mental healthcarE (PRIME), a project supporting delivery of mental healthcare integrated into primary care in a rural district in south central Ethiopia. In-depth interviews (*n* = 21) were carried out with stakeholders selected purposively from mental health service users, caregivers, community leaders and healthcare administrators. A focus group discussion (*n* = 12) was conducted with community health extension workers. Framework analysis was employed using an adapted version of the access framework developed for use in contexts of livelihood insecurity, which considers (1) availability, (2) accessibility and affordability. and (3) acceptability and adequacy dimensions of access.

**Results:**

Primary care-based mental healthcare was considered as a positive development, and would increase availability, accessibility and affordability of treatments. Low levels of community awareness, and general preference for traditional and religious healing were raised as potential challenges to the acceptability of integrated mental healthcare. Participants believed integrated mental healthcare would be comprehensive and of satisfactory quality. However, expectations about the effectiveness of treatment for mental disorders were generally low. Threats to equitable access to mental healthcare were identified for perinatal women, persons with physical disability, those living in extreme poverty and people with severe and persistent mental disability.

**Conclusion:**

Establishing an affordable service within reach, raising awareness and financial support to families from low socioeconomic backgrounds were suggested to improve equitable access to mental healthcare by vulnerable groups including perinatal women and people with disabilities. Innovative approaches, such as telephone consultations with psychiatric nurses based in nearby towns and home outreach need to be developed and evaluated.

## Background

Neuropsychiatric disorders contribute to 7.4 % of the total global burden of disease [1]. When considering years lived with disability, mental disorders make the second highest global contribution [1]. Despite the substantial disability and high rates of premature death due to mental disorders, the current level of public policy attention and care access is extremely low, especially in countries of sub-Saharan Africa [2, 3]. Globally, it is estimated that 50 % of people with severe mental disorders remain untreated and the treatment gap in low-income countries (LICs) is as high as 90 % [4, 5].

In response to this high treatment gap, the World Health Organisation (WHO) has introduced the mental health gap action programme (mhGAP); a framework for scale-up of mental health services for selected priority disorders through integration into primary care [6]. The mhGAP intervention guide is a contextually adaptable, evidence-based set of packages of care suitable for delivery by non-specialists in low resource settings.

Developing integrated services that are accessible to all requires rigorous understanding of the barriers experienced by disenfranchised sections of society. The concept of access to healthcare is complex as it incorporates factors beyond availability of the service [7]. Poor living conditions, place of residence, history of previous access, low level of education, inequitable access to power and economic resources could be some of the social determinants of access to healthcare [8–11].

Ethiopia has a healthcare system focusing on disease prevention in the primary care setting [12]. Healthcare in the country is characterised as under-resourced and, at the present time, can only be accessed by the majority of the population through out of pocket funding [13]. Mental health conditions lead to increased financial stress as the condition causes chronic disability and requires long-term access to medications and consultations [14]. Therefore, equitable access to mental healthcare in Ethiopia is complicated by poverty, weak infrastructure and the limited numbers of facilities providing mental healthcare, most of which are situated in or around big cities. Recent initiatives seek to expand access to mental healthcare through integration with primary healthcare [15, 16] but little is known about how equitable access to mental healthcare can be ensured.

The overall aim of this study was to inform the development of equitable and accessible mental healthcare integrated into primary care services for people with severe mental disorders in rural Ethiopia [17]. Specifically, we sought to identify the potential barriers to equitable access to care to mental healthcare and how they might be overcome.

## Methods

### Study design

A qualitative study.

### Study setting

The study was conducted in Sodo district, one of the 15 districts in the Gurage zone of the Southern Nations, Nationalities and Peoples Regional state, located around 100 km from Addis Ababa. The total population of Sodo district is about 170,000 [18]. Sodo district was selected because it is reflective of the broader agroclimatic, geographical and social dynamics of the country and is the location of the Programme for Improving Mental health care, a multi-country initiative to implement, evaluate and scale-up primary care-based mental healthcare [16, 17]. Sodo district neighbours Butajira, the site of longstanding mental health and public health research, thus providing an infrastructure from which to operate. The main inhabitants of the district are the Gurage ethnic group and most people are followers of Orthodox Christianity (97 %). Sodo district has diverse agro-ecological zones including highlands and lowlands, with an elevation of 1500 m above sea level. The district is characterised by difficult terrain, a lack of reliable means of transportation and limited reach of all-weather roads. Carts, motorbikes, lorries and minibuses are the only available forms of transportation, with no fixed departure time, stops, or defined routes. Transportation fares are mostly negotiable and subject to abrupt hikes particularly if the person traveling has a serious health problem, including mental illness, or if it is an emergency.

### Mental healthcare context of the district

Historically, biomedical care for people with mental disorders has not been available in the Sodo district. Help for people with severe mental disorders has, therefore, either been sought from a psychiatric nurse-led out-patient clinic in the neighbouring district (30–50 km away) or from traditional and faith healing sites located within and outside the community [19, 20]. As part of PRIME, general primary care workers will be trained to deliver basic mental and neurological healthcare for priority disorders: psychosis, depression, epilepsy and alcohol use disorders [17].

### Population and sample

#### Sampling

The sample from this study was nested within the broader qualitative study conducted for the PRIME formative study, focusing on stakeholders from the community and district level administration [21]. For community participants, inclusion criteria were communicated to the health extension workers (community health workers with 1 year training in preventive care), who then facilitated identification of representative participants from different segments of the community. As there was no mental health service in the district at the time of this study, the service user and caregiver interviews were conducted with people selected from the neighbouring district who were accessing care through a hospital based, psychiatric outpatient clinic. The neighbouring district shares similar socioeconomic and cultural characteristics with the study site.

### Study procedures

#### Data collection

In-depth interviews were conducted with the majority of respondents as they represented disparate groups within the community (see Table 1). One FGD was conducted with the health extension workers as they formed a homogeneous group (*n* = 12). In-depth interviews with community members and district administrators were held in a neutral venue, a meeting room in one of the hotels in the district. Interviews with mental health service users and their caregivers were carried out in or around their residence, based on their preference. The FGD was conducted in a primary healthcare centre located in Bui, the main town in Sodo district. The official language of Ethiopia, Amharic, was used throughout. All of the interviews and the FGD were audiotaped. In-depth interviews lasted from 45 to 90 min while the FGD took around 100 min. Two people, a facilitator and a note taker, facilitated the FGDs. The data collectors were three Ethiopian women with a Masters degree in Social Work and two of them are enrolled in a PhD programme supported by the PRIME project (MH and MS). All of the data collectors have proven track record of conducting qualitative interviews in the district and are familiar with the social and cultural makeup of the community. Data were collected in two phases, the first batch in 2012 and additional interviews (with caregivers and mental health service users) in 2015.

**Table 1:** Socio-demographic details

Mode of data collection	Participant type	Gender		Age	Other characteristics
		Male	Female		
FGD	HEWs	0	12	25-38	One year training after completing grade 10 or 12
In-depth interviews	Service users	3	3	26-50	All rural residents, non-literate
	Caregivers	3	2	35-60	Rural residents, non-literate
	Traditional and faith healers	3	0	36-65	Herbalists, holy water attendants
	Community leaders	4	0	50-65	4^th^ -8^th^ grade
	NGO representatives	2	0	32, 33	First degree
	District health office representative	1	0	-	First degree

**Fig. 1:**
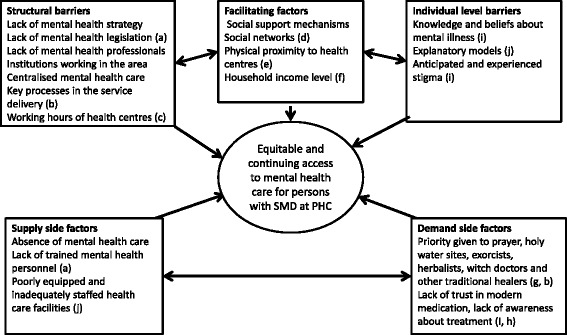
Conceptual framework

Prior to the fieldwork, topic guides were adapted from the PRIME cross-country guide and checked against a pre-existing framework for conceptualising healthcare access which was developed for low-resource settings [22]. The five dimensions of access were grouped into three major themes (1) availability, (2) affordability and accessibility and (3) acceptability and adequacy. The regroupings were done due to the interrelationships witnessed. The revised dimensions have been found to influence help-seeking for illness [22]. These dimensions comprise both the supply-side and demand-side aspects of access to healthcare. In additional file 1, the dimensions of access are presented as conceptualised in this study. While the availability, accessibility and affordability dimensions deal with factors affecting potential access, the acceptability and adequacy dimensions are mainly concerned with issues pertaining to actual access. Given the limited baseline access to mental healthcare, the latter were adapted to refer to anticipated barriers in adequacy and acceptability. Further elaboration of the framework was based on the Ethiopian literature. Accordingly, factors such as household size, educational level, history of previous access and exposure to media are factors known to determine access to general healthcare [23, 24]. Additionally, access to the existing mental healthcare services in Ethiopia is known to be seriously constrained by various factors, including financial problems, preference for traditional care, seasonal roads, distance to health facility, high cost of care and perceived stigma affect one’s decision to access mental healthcare [25–27]. See Fig. 1.

### Data analysis

In-depth interviews were transcribed in Amharic and translated verbatim into English. Data were analysed using OpenCode version 4.02, an open source programme to support management and analysis of qualitative data [28]. Framework analysis [29, 30], an approach to qualitative data analysis used increasingly in the areas of healthcare and policy analysis, was employed during data collection and analysis. Codes from the data were generated and further categorised into the different dimensions of access based on a pre-existing framework (see additional file 2). MH did the initial coding of all the interviews and the FGD. CH reviewed lists of codes and checked consistency of codes against the data and the framework. MH and CH discussed, adapted the framework and agreed on the final codebook for analysis.

## Results

### Sociodemographic characteristics of participants

In-depth interviews were conducted with service users (*n* = 6), religious and faith leaders (*n* = 4), a district health office representative (*n* = 1), a herbalist (*n* = 1), community elders (*n* = 2), representatives of non-governmental organisations (*n* = 2) and caregivers of people with severe mental disorders (*n* = 5) (Table 1). Findings are presented according to the dimensions of access as contextualised for this study. Factors affecting equitable access to mental healthcare are presented separately.

- Theme I: Availability
  - Most participants spoke of the current absence of a service in the district as one of the biggest barriers to accessing mental healthcare, affecting decisions about where to seek care and when to initiate help seeking. At present, only those with sufficient financial resources are able to access mental healthcare by travelling to distant places:
  -
    *If treatment starts [in the district], I can’t explain how happier I would become. Instead of travelling to far places in search of treatment and spending too much money and lose their assets in the process, it would be very good if they are treated here. People go to Amanuel [a psychiatric hospital in Addis Ababa] and other places including holy water (tsebel) places like Shenkora….People travel to different countries, regions to receive treatments. *04_community elder
  - The plan to bring mental health closer to the community was expected to overcome the financial and logistical barriers associated with distant travel for many people:
  -
    *…there is no service at the health centre level. Many people are affected by the problem because there is no treatment or counselling at the health centre level. Having the service at the local level is invaluable. Mental illness is a big problem that degrades people…..Someone who cannot afford to go to Addis Ababa for treatment will [be able to] access the service.* 05_religious leader, Orthodox church
  - Participants suggested that making mental health services locally available would enhance the quality of life, functioning and productivity of people with severe mental disorders. It was also mentioned to reduce human rights abuses such as physical assualts and shackling. Restraining a person with mental illness, for example with chains, was reported to be a common response to the lack of accessible care. Participants also remarked that people in traditional healing places were mostly restrained when being brought to, or while attending care at traditional and faith healing sites.
  - In the absence of mental health services, participants reported that people tried different avenues to find solutions for their situations. Costs associated with seeking treatment from traditional healers, including payment for gifts, traditional medicines, travel expenses and other forms of contributions were reported to be much higher than would be incurred if people were able to access modern care.
- Theme II: Affordability, Accessibility
  - Participants reported sets of interwoven potential barriers to access to mental healthcare in a primary care context. Among these were challenges with leveraging social support, the severity and persistence of illness and difficulty conveying a person with severe mental disorder. Reachability of the service in terms of geographic proximity to one’s area of residence was one of the key recurring themes across the interviews. Increased accessibility and better affordability of mental health services were said to result in better help-seeking behaviour. Shorter distance travelled was also said to increase affordability due to the decline in the indirect costs of treatment.
  - It was indicated that inability to pay for treatment or associated expenses is considered as one of the major potential barriers to accessing care:
  -
    *“If the family is poor it is very difficult to get treatment. I guess it is clear. It is poverty. Unless it is financial problem who would ever like to keep his/her family at home with such a suffering? It is lack of money that hinders people from getting treatment”.* 02_traditional healer
  - The other argument by some of the participants is that the provision of mental healthcare in the local setting would address affordability concerns. Establishing the service within reach implies reduction in the indirect costs of treatment such as transportation, meal and accommodation.
- Theme III: Acceptability and adequacy
  - Excitement about the planned service was evident across the interviews. Previous encounters with mental health services and level of awareness about treatment of mental disorders were mentioned to be important predictors of acceptability. People with no previous experience of modern mental health services were said to have potential difficulty to accept health service-based care. Causal attribution of mental illnesses was underlined as an essential factor affecting help-seeking. The majority of participants considered poverty, thinking too much, anguish and adverse life events to be the causes of mental disorders. There was also a strong belief in the community that some mental illnesses were attributable to evil spirits or demonic possession.
  -
    “*Some spirit that babbles as ‘I’m Satan, or I’m a magic spirit’, a spirit that makes them feel dizzy and mentally strained would go away*”. 08_holy water attendant
  - The protestant church leader also confirmed this stating that some forms of mental disorders are caused by evil spirits and are healed only through prayer and exorcism. According to him, due to such overarching beliefs, people tend to seek care first at traditional healers and may not find healthcare-based treatment to be acceptable. The HEWs also emphasised that these beliefs were shared by majority of the population. However, the impact of having such beliefs on help-seeking is not discussed in the in-depth interviews.
  - Various explanatory models of illness were mentioned across all the community representatives. In some cases, it was likely that one person might hold more than one explanation as to how and why illness started. One’s fate, curse, wearing perfumes during the sun, worries about life/school and falling in love were some of the explanations given to cause mental disorders. It was reported that people who hold these explanatory models strongly believe that traditional healings are more suitable than modern treatments.
  - Although this could be partly linked with non-availability of modern treatment in the district, people tended to go to places as far as 300kms away from their residence to attend traditional healings. The HEWs focus group discussion also shed light into the importance of awareness of families and acceptability of services. How these two affect the decision where to seek care from is illustrated below:
  -
    *There is awareness problem in the community. This means instead of coming to health centres, the community prefers to go to holy water [tsebel] and other places. They think that tsebel is effective than modern medication. If we start service for mental illness, this may have some good thing for our community. But as our health centres do not have medication, the community may not trust them and seek service.* HEW_FGD
  - The decision to seek help from traditional healers could be related to lack of trust in modern medication or absence of any modern mental healthcare providers in the vicinity. Although the need for comprehensive healthcare was acknowledged by many, lack of awareness from the community’s side is presented as a potential barrier to access:
  -
    *Health by itself is a manifold issue and mental health treatment is one of it. I don’t think our community would be against having these services. They’d prefer to have it near to them. But the thing is our community has its traditions, so a lot of work should be done. Awareness creation should address this. There are people who go through a lot of stress and lose their mind. So in light of redeeming these people’s lives it will be very essential to have these services and enabling the community to understand and utilize it.* 10_preacher in protestant church
  - Another caregiver of a person with severe mental disorder also strengthened the above claim as:
  -
    *For the first two years, we thought it was an evil spirit and sought for traditional help. Around the end of the second year, we took him to modern treatment…..*13_Caregiver
  - Such attitudes were reported to be some of the potential barriers to access. The belief that mental illness is not curable contributes to the low priority given to modern medication. The health extension workers also mentioned that many families opt to seek care at traditional care providers first before they consider travelling to modern facilities.
  - The participants did not prioritise concerns related to the adequacy of a primary care-based service. Availability of the service within the community was perceived to encourage early access as people would get the opportunity to witness changes in the lives of those who had been treated. The decision to access services was said to be largely influenced by the success or failure of those who had previous experience with mental health facilities. The HEWs also indicated that success stories of those treated will wipe away doubts regarding effectiveness of modern care.
  - According to the community elders, the number of people treated by the programme and the impact of treatment on the lives of those treated would be considered to be the most important indicator of quality of care. Two of the religious leaders disclosed their fear that the quality of care could be compromised if the service was provided at the health centre level. This was also shared by another participant below:
  -
    *From our experiences so far, when [services] are decentralised, we face problems with maintaining the quality. This may be in terms of the health professionals; the follow up. There are instances where they neglect their responsibilities. The person with a mental illness may not get the proper treatment. This is my concern. Such problems may be faced at this level*. IV11_NGO representative 2
  - The above argument is based on the current lack of training and absence of psychotropic medications at the health centre level. Hence, referrals to the nearby psychiatric hospitals, supervision by trained psychiatrists and consultations with existing primary healthcare workers with better training in mental healthcare was recommended to enhance quality.

### Equitable access

The possibility of differential access to care along the lines of gender, physical disability, area of residence and socioeconomic status was indicated in many of the interviews. The HEWs considered urbanity, the male gender, better socioeconomic status and education as proxy of access to mental health literacy and treatment. People from the lower economic stratum were said to have difficulty accessing care although the service is locally available.

*If there is a pregnant woman or if there is someone who is physically weak, accessing services may be difficult. If they do not have money, that will make it even difficult. If you do not have money, you can access nothing. It is not because the person is a woman or a man. The main thing is having money. If they do not have money, they cannot benefit from the service being provided.* 05_religious leader Orthodox church

Although the community elders were confident about the prospect of leveraging social support to those unable to pay, the rest of the participants were very reserved regarding the community’s role. Some rare cases in which some community associations helped people with severe mental disorders were mentioned during few of the interviews. However, caregivers of people with severe mental disorders reported that it was very difficult to get support from the community. The ability to draw upon social support was reported to decline if the condition causes prolonged disability and severe functional impairment. Some of the participants highlighted that obtaining social support gets tougher as years pass by:

*Different households have various problems. They may not be able to cover the treatment costs due to poverty. The other problem is that it is a long-term illness. People will get tired of you if you are ill for longer period of time. Your relatives could help you till you get well. But, people will get tired of you when you have an illness that stayed with you for years.* Female service user

The community members also stated that one’s capacity to reciprocate determines the social capital that the person could access to pay for on-going care. Some of the interviewees were optimistic about the affordability of services. According to one of the community elders,

“*As long as the service being provided is in government health centres, it is affordable for most people as the government also subsidizes it. We do not have that poor people who fail to access the service at health centre level”.* 03_community elder

The above assertion was not endorsed by everyone. Some of the participants disclosed their fear that the cost of medication could keep some people away. Although primary care facility based services were believed to enhance affordability, it was stated that poverty poses significant burden especially when the condition causes chronic disability requires long-term care. Interview with the district health office head confirmed that the government has introduced fee waivers for community members who are considered ‘poorest of the poor’. Nevertheless, service users and caregivers reported that obtaining the poverty certificate (certificate declaring eligibility for fee waivers) is often a challenge or they still have to pay for some medications.

On the other hand, the HEWs stated that minimum fees should be in place even for those who are considered ‘poorest of the poor’.

*“I do not believe in giving the medication for free. It should consider the economic background of families. It will be effective this way. The community does not appreciate free medication. Even when we give them vaccinations for free, they would consider it effective when they spend some money.”* HEW_FGD

Some of the HEWs however, disagreed saying that the whole package of treatment should be given for free. Nevertheless, majority of the FGD participants believed that complete abolition of fees would result in less regard to the effectiveness of the treatment.

Although the HEWs mentioned physical disability as a barrier to access, the community members considered it as less important. Financial strength of the family and the ability to leverage social support was mentioned as key to access.

*I don’t think so [physical disability is a problem] because mentally ill people are brought to healthcare facility by other people in most cases. We can take this one as a factor that hinders them from coming to the service for example if you don’t have money for transport it is very difficult to take your ill relative to hospital.* 07_NGO representative

Accessibility was also reported to have a specific gender dimension. When women attempted to access care away from their residence, sometimes they might pay unbearable costs in relation to childcare as stated below.

*I was breastfeeding when I first went for treatment. My neighbours were kind enough to breastfeed my baby who I left behind. I was mentally ill. I was not willing to breastfeed or take care of him. All breastfeeding women in my neighbourhood took turns breastfeeding my baby. But the baby did not survive because he was breastfed by other people and was also malnourished. Had there been treatment in the nearby, my baby would have survived.* Female service user, 01

HEWs also underlined the challenges experienced by perinatal women. The HEWs training has contents that remarks pregnant women should not be given any medication throughout the pregnancy. As one of the participants stated, “*We the HEWs prevent them from using any tablet during pregnancy. Therefore, they may possibly think taking tablets including those taken for treating mental illnesses are wrong*”. There is a fear that lack of awareness of the HEWs on how mental healthcare delivery works in relation to pregnant women might create potential complications in access to care in this group.

Concerns related to fear of being raped or assaulted on their way to treatment facilities was also mentioned to affect women’s access to treatment. As is the case in most parts of rural Ethiopia, people have to travel long distance on foot which mostly puts women and vulnerable groups in greater risk of sexual violence. Some of the FGD participants also stated that women may be hesitant to go to care due to household chores or childcare priorities. The HEWs reported that families prefer putting women in chain than taking them for treatment.

*If a woman suffers from psychosis or depression, both the community and her family may think that she should remain at home. They may think that it is costly to take her to treatment places. Therefore, they will put her in chain at home. They may think she should stay at home because staying at home does no harm to the woman or they may also take her to different worship places. They may go to tsebel places. Therefore, taking such alternatives may make treatment a difficult task.* HEW_FGD

The above factors are believed to cause disproportionate representation of women, the economically poor and people with disabilities in those who did not access care.

### Strategies to overcome barriers

The participants recommended the approaches and strategies detailed in Table 1 to overcome the potential barriers to accessing care.

## Discussion

### Availability

We have learned that the absence of mental healthcare in the district contributed significantly to delayed help-seeking and non-engagement with care. The majority of participants indicated that families opt to seek traditional help for their member with mental disorder as indicated in a previous population-based quantitative survey [20].

In this qualitative study to inform mental health service planning in a rural Ethiopian district, the integration of mental healthcare into primary care was anticipated positively by stakeholders. Stakeholders maintained high expectations in terms of improving access to care, reduce delays in obtaining treatment and improving quality of life for people with severe mental disorders.

### Affordability and accessibility

Affordability was seen as the main on-going barrier to accessible care, even when locally available, because of expenses associated with long-term treatment. Although the monthly costs might look insignificant, long-term care poses a significant financial burden on families in this subsistence farming community, particularly as their ability to leverage social support declines over the years [21]. Healthcare planners tend to rely on Euclidean distance rather than taking into account the complexities of topography or the scarcity of means of transportation [31]. Findings of this study elucidate that factors beyond straight-line distance define the perception of accessibility. For example, conveying the person to health facility poses a tremendous challenge when the person is acutely disturbed or where the carer is a female or physically weak.

In all of the interviews, it was said that accessibility of services is related to increased ability to pay due to reduction in indirect costs of treatment. In some of the interviews it was indicated that the service being available will increase acceptability since there will be success stories coming out from the service.

Interventions to address poverty and raise the living standards of families are essential to ensure equitable access to care for families experiencing financial challenges [32]. For improved mental health outcomes, mechanisms to bring about improvement in clinical outcomes for persons with severe and persistent mental disorders should go parallel with economic interventions [32]. Outreach programmes, community mental health awareness campaigns and clinics located in renowned places of traditional healing should be considered.

### Acceptability and adequacy

Acceptability of the new service was considered a potential barrier due to differing attributions for mental health problems. Adequacy of a primary-care based service was not a priority concern. Although the importance of cultural beliefs often distracts planners, having the financial resources was repeatedly indicated to be the deciding factor for access. Nevertheless, given the poor transportation infrastructure in the district, experiences show that even those who are able to pay may not be able to access public transportation due to stigma and discrimination towards people with mental disorders.

The salience of sociocultural barriers appeared to have been eclipsed by barriers exclusively pertaining to financial access in the narratives of respondents. The extent to which this is truly the case can be evaluated empirically when PRIME makes biomedical mental healthcare affordable and accessible geographically.

### Equitable access

The new model of integrated mental health in the primary care setting is likely to be important to address the issue of equitable access. The approach takes into account context and is sensitive to barriers to equitable access by identifying bottlenecks from the outset. Settlement patterns, configuration of health facilities and access to a reliable means of transportation are key to ensuring equitable access to mental healthcare.

People from low socioeconomic backgrounds, those with physical disability or frailty and women were considered to be potentially disadvantaged, with the need to adapt the primary care-based model in order to ensure an equitable service. Strategies to overcome challenges to equitable access to mental healthcare are summarised in Table 2.

**Table 2:** Summary of barriers and strategies

Barriers	Recommended strategies	Specific interventions
Theme I: Availability		
Absence of mental health services	Making the service locally available	Integration to primary care
Absence of psychotropic medications	Ensure availability of medications	Include psychotropic medications in the drug list
Theme II: Affordability and accessibility		
Inability to cover treatment related costs	Providing cheaper treatment	Financial support to ‘poorest of the poor’
		Subsidising mental health treatment
Longer distance travelled to access services	Establishing the service within reach	Facilitate emergency transportation
		Plan outreach mental health service
Indirect expenses of seeking care		Interventions to improve household income
Theme III: Acceptability and adequacy		
Causal attribution: attribution to some evil spirits	Raise awareness	Involving HEWs in raising awareness and initiating discussions
		Engaging religious leaders in awareness raising
Lack of trust in modern medication	Raise awareness	Involving service users who recovered
		Telling success stories of those treated
Lack of awareness (the belief that mental illnesses are not curable)	Raise awareness	Engage traditional healers in educating the community
		Amplify success stories from treatment
		Engage service users who recovered in assisting HEWs in their awareness raising endeavours
High regard given to traditional healing	Raise awareness	Train traditional healers on detection of core symptoms of mental disorders
		Create referral linkage framework
		Work with church leaders
		Mainstream mental health contents in community conversations
Concerns about quality of the service	Supervision	Supportive supervision by psychiatric nurses
		Telephone consultations

The majority of participants did not endorse gender as an important barrier to access. Some of the participants dismissed the claim that women lack wherewithal to make adequate cash for treatment, even though previous studies have indicated low financial empowerment of women in rural Ethiopia [33]. In our study, little was mentioned about the femininity of poverty with respect to access to financial resources. Nevertheless, the female HEWs emphasised the unique challenges that women are confronted with in when they try to access care with a focus on perinatal women. There is also evidence that perinatal women are at risk of discontinuing the treatment [34]. Due to the limitations in training of primary care workers, the WHO mhGAP intervention guide recommends the referral of perinatal women to specialised providers [35]. Nevertheless, there is a fear that this may result in perinatal women being unable to access care at all. Being referred to a hospital necessitates additional travel or accommodation costs which some of these women may not find affordable. To promote access in this group, innovative approaches like telephone consultations with psychiatric nurses might help.

### Limitations

Of the 22 in-depth interviews, only five were women. This is mainly because the patriarchal community structure where mainly the men hold majorities of the leadership positions. To understand the issues of women, we involved 12 female HEWs in the FGD as they work closely with women. We did not obtain feedback from our participants about the emerging findings with respect to our framework.

## Conclusion

Understanding the barriers to access to mental healthcare in a primary care setting from the outset significantly enhances the focus on equity. The approach helps to identify vulnerable groups like people with disabilities and perinatal women who might experience unique challenges to access care. Establishing an affordable service within reach, raising awareness and financial support to families from low socioeconomic backgrounds were suggested to improve equitable access to mental healthcare in this group. The introduction of innovative approaches such as telephone consultations with psychiatric nurses based in the nearby towns was also mentioned essential. We hope that the next step would be to evaluate whether the programme succeeds in providing equitable access to mental healthcare from the dimensions of treatment initiation, retention in care and benefit from care.

## Abbreviations

FGD, Focus Group Discussion; HEW, Health Extension Worker; mhGAP, mental health Gap Action Programme; NGO, Non-Governmental Organisation; PRIME, PRogramme for Improving Mental health carE; WHO, World Health Organisation
